# The influence of temperature on the photoluminescence properties of single InAs quantum dots grown on patterned GaAs

**DOI:** 10.1186/1556-276X-7-313

**Published:** 2012-06-19

**Authors:** Juha Tommila, Christian Strelow, Andreas Schramm, Teemu V Hakkarainen, Mihail Dumitrescu, Tobias Kipp, Mircea Guina

**Affiliations:** 1 Optoelectronics Research Centre, Tampere University of Technology, Korkeakoulunkatu 3, Tampere, FIN-33720, Finland; 2 Institute of Physical Chemistry, University of Hamburg, Grindelallee 117, Hamburg, 20146, Germany

**Keywords:** III-V semiconductors, InAs, Quantum dots, Site-controlled quantum dots, Molecular beam epitaxy, Nanoimprint lithography, 78, optical properties, condensed-matter spectroscopy and other interactions of radiation and particles with condensed matter, 78.67.-n, optical properties of low-dimensional, mesoscopic, and nanoscale materials and structures, 78.67.Hc, quantum dots

## Abstract

We report the temperature-dependent photoluminescence of single site-controlled and self-assembled InAs quantum dots. We have used nanoimprint lithography for patterning GaAs(100) templates and molecular beam epitaxy for quantum dot deposition. We show that the influence of the temperature on the photoluminescence properties is similar for quantum dots on etched nanopatterns and randomly positioned quantum dots on planar surfaces. The photoluminescence properties indicate that the prepatterning does not degrade the radiative recombination rate for the site-controlled quantum dots.

## Background

Single semiconductor quantum dots (QDs) are the building blocks for future information processing platforms, such as quantum cryptography and quantum computing [1-3]. QDs have been exploited as single [4,5] and entangled photon sources [6,7]. In order to enable such applications, the QDs should be fabricated at well-defined positions, rendering impractical the standard epitaxial processes that would result in randomly positioned QDs. The control of QD position can be achieved by creating preferential nucleation sites via patterning the deposited surface. Site-controlled quantum dots (SCQDs) have been fabricated utilizing various patterning techniques [8-12] combined with molecular beam epitaxy (MBE) or metalorganic vapor phase epitaxy. The optical properties of single QDs are easily deteriorated by defects, which are induced during the patterning and subsequent overgrowth. The defects cause nonradiative recombination channels, which degrade the QD photoluminescence (PL). Measuring the optical properties of a QD as a function of temperature provides a method to assess the quality of the QDs.

In this paper, we study the temperature-dependent PL of single SCQDs fabricated on a nanoimprint lithography patterned GaAs(100) surface. We compare their PL properties to the ones of single self-assembled quantum dots (SAQDs) grown on unpatterned surface.

## Methods

The InAs QD sample was fabricated on a GaAs(100) substrate by combination of MBE and soft ultraviolet nanoimprint lithography (UV-NIL). First, a GaAs buffer layer, an AlGaAs barrier layer and a GaAs layer were grown by MBE. Second, the sample was patterned *ex situ* by UV-NIL using mr-UVCur06 (Micro Resist Technology GmbH, Berlin, Germany) as an etch mask and an EVG-620 (EV Group, St. Florian am Inn, Austria) mask aligner. The patterned area consisted of holes with a diameter of 100 nm arranged in a square lattice with the period of 1.5 μm. After patterning, the sample was chemically cleaned, and the native oxides were removed using IPA-, HCl-, and NH_4_OH-based solutions. The process is described in more detail in [13]. After chemical treatment, the sample was loaded into the MBE reactor, and a 30-nm GaAs buffer layer was grown at 470°C. Growth-interrupted MBE [13,14] at 540°C was used to form single SCQDs in the patterned holes and low density SAQDs randomly positioned outside the patterned area. Finally, the QDs were capped by 20 and 50 nm of GaAs grown at 540°C and at 590°C, respectively, and 50-nm AlGaAs grown at 590°C. The structure was finished by a 10-nm GaAs layer.

Micro-PL (μPL) measurements were performed at various temperatures up to 70 K using a continuous-wave laser emitting at 532 nm for excitation and a microscope objective (numerical aperture = 0.8) for diffraction-limited laser beam focusing and PL light collection. The PL signal was dispersed by a 50-cm spectrometer containing a 1,200 lines/mm grating and detected by a cooled Si CCD camera. The spectral resolution of the setup was 66 μeV.

## Results and discussion

**Figure 1:**
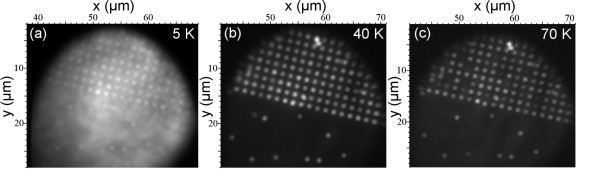
**PL intensity images from the edge of the patterned area.** Intensity within the spectral range of 850 to 1,000 nm was measured at temperatures of 5 K (**a**), 40 K (**b**), and 70 K (**c**). The excitation power and wavelength were 1.77 μW and 532 nm, respectively.

In order to evaluate the large scale optical quality of the sample, we added a diverging lens into the beam of the excitation laser in the μPL setup. The widened illumination field had a diameter of about 30 μm on the sample. Figure 1 shows PL intensity images from the edge of the patterned area within the spectral range of 850 to 1,000 nm at temperatures of 5, 40, and 70 K. Regularly spaced (1.5-μm period) bright spots illustrate PL emission from single SCQDs. Spots outside the array originate from SAQDs. For the lowest temperature (Figure 1a), the whole PL image is affected by a relatively strong background. Spectrally resolved measurements confirm its origin in the wetting layer (WL) emission, which is typical for low density QDs [15]. At low temperatures, the photoexcited carriers are trapped and recombined in the WL at localizing potentials induced by irregularities in WL [16]. The effect was confirmed by the fine structure of the WL PL (not shown). At 40 K (measurement shown in Figure 1b), the emission from WL is reduced because of the thermal escape of the carriers from the localizing potentials and a very thin WL typical for this growth technique [17]. An array of intense PL spots from the single SCQDs with 1.5-μm period is observed. When the sample was heated up to 70 K (measurement shown in Figure 1c), the PL emission from some individual QDs decreased due to thermal escape of carriers from the QD states and decreased carrier population in the WL acting as a carrier reservoir for QDs [16]. In spite of the slight deviations in uniformity, most of the SCQDs exhibit bright luminescence even at relatively high temperatures, indicating a low amount of nonradiative recombination processes. 

**Figure 2:**
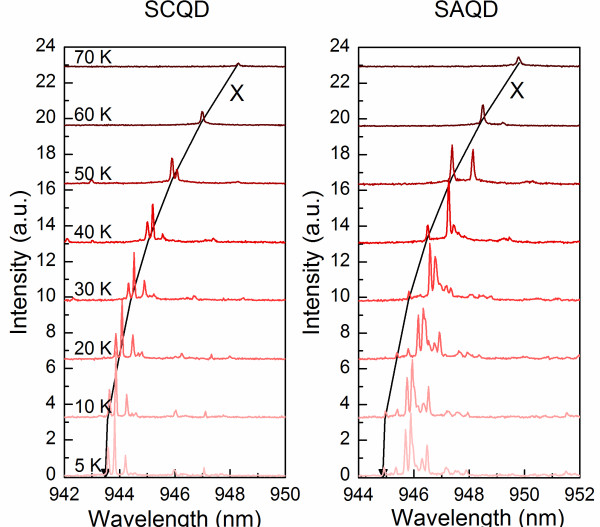
**Single QD emission spectra for SCQD (a) and SAQD (b) at temperatures 5 to 70 K.** The spectra are vertically shifted for clarity. The excitation power was 2 nW.

**Figure 3:**
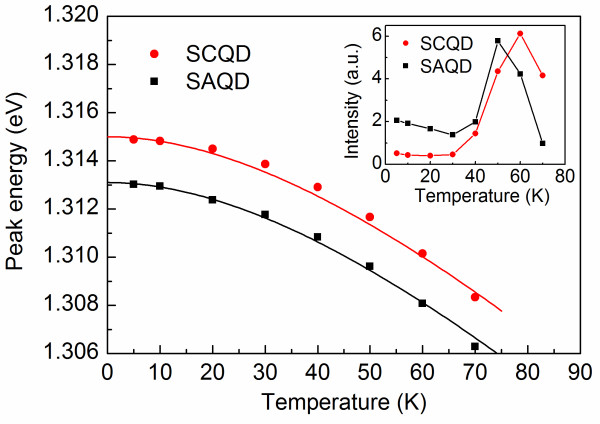
**Peak energies of the exciton (X) transitions as a function of temperature.** Solid lines indicate the slope of the temperature dependency of the InAs band gap energy obtained by Varshni's law. The parameters are *α* = 0.27 meV/K and *β* = 135 K. The inset shows the integrated PL intensities of the exciton transitions.

**Figure 4:**
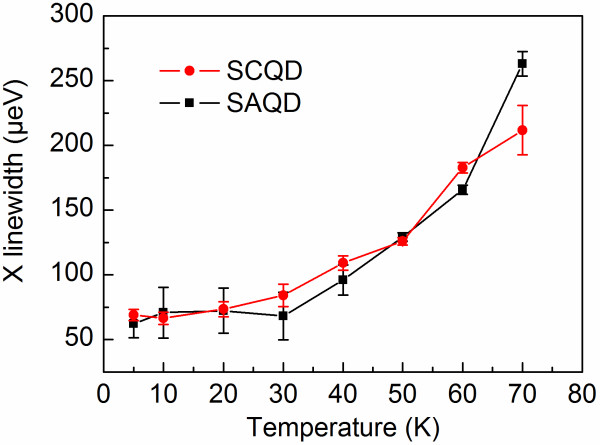
**Linewidths of the exciton transitions as a function of temperature.** The linewidths are obtained from Lorentzian fits to the exciton peak. Error bars indicate the error of Lorentzian fits.

Further on, we performed μPL measurements with focused excitation within a temperature range of 5 to 70 K for randomly selected bright SCQDs within the pattern as well as for SAQDs outside the patterned area. Figure 2a,b shows typical sets of single QD emission spectra of a SCQD and a SAQD, respectively, at temperatures from 5 to 70 K. At high temperatures, only single exciton (X)-related peaks are visible in the spectrum, whereas at lower temperatures, also emission from biexciton and charged exciton states is observed. Figure 3 shows the peak energies of the exciton transitions (marked with X in Figure 2) for SCQD and SAQD as a function of temperature. The energies obey the temperature dependency of the InAs band gap energy as obtained by Varshni's law (solid lines), which is shifted in energy [18-20]. The integrated intensities of the single exciton PL for SCQD and SAQD are shown in the inset of Figure 3. The increase in intensity around 50 K is attributed to the increased amount of carriers transferred to the QD from the WL [16]. Figure 4 shows the linewidth of the exciton transition as a function of temperature calculated from Lorentzian fits to the exciton peak. The exciton linewidths for SCQD and SAQD show very similar temperature dependencies. At low temperatures, i.e., 5 to 30 K, the linewidth appears to be limited by the spectral resolution of 66 μeV of the measurement setup, but it increases rapidly to 250 μeV when increasing the temperature from 40 to 70 K. The linewidth broadening is mainly caused by phonon scattering [21] and is in good agreement with previous results for SAQDs [22] and SCQDs [23,24]. 

## Conclusions

We have studied the influence of temperature on the PL properties of single site-controlled InAs QDs fabricated by a combination of soft UV-NIL and MBE. We have shown that the QDs in etched holes emitting at 945 nm have temperature-dependent PL properties similar to the self-assembled QDs grown on planar surface. The PL properties indicate that the defects induced by the patterning do not degrade significantly the emission of the site-controlled QDs. Thus, soft UV-NIL-positioned QDs are considerable candidates for fabricating large-scale optoelectronic devices.

## Abbreviations

MBE: Molecular beam epitaxy; PL: Photoluminescence; μPL: Micro-photoluminescence; QD: Quantum dot; SAQD: Self-assembled quantum dot; SCQD: Site-controlled quantum dot; UV-NIL: Ultraviolet nanoimprint lithography; WL: Wetting layer; X: Single exciton.

## Competing interests

The authors declare that they have no competing interests.

## Authors’ contributions

JT carried out UV-NIL patterning, participated in design and data analysis, and drafted the manuscript. AS and TVH carried out the MBE growths and participated in design. CS and TK performed the PL measurements. MD and MG conceived of the study and participated in its design and coordination. All authors read and approved the final manuscript.
